# 

**Published:** 1900-04

**Authors:** 


### CONTENTS.

ORIGINAL COMMUNICATIONS. PAGE

Common-Sense Occlusion, or "Bite." By W. WARRINGTON EVANS, M.D., D.D.S., Washington, D. C. . . . 221

Dental Notes. By WILLIAM ROLLINS, Boston, Mass. . . . 235

Notes on Silver Nitrate. By DR. J. MORGAN HOWE, New York . . . 237

Education in Dental Medicine. By HAROLD WILLIAMS, M.D. . . . 239

Dentistry is to the Eye that sees it as to that Eye it seems to be. By DR. A. J. FLANAGAN, Springfield, Mass. . . . 245

ABSTRACTS AND TRANSLATIONS.

On the Rôle of Systemic Hyperacidity, and of Sulphocyanides in the Saliva, in Chemical Abrasion of the Teeth. By M. MICHAELS . . . 248

REPORTS OF SOCIETY MEETINGS.

The New York Institute of Stomatology . . . 256

American Academy of Dental Science, Annual Meeting . . . 275

EDITORIAL.

The Decision of Judge Townsend . . . 277

Special Notice . . . 279

BIBLIOGRAPHY . . . 279

DOMESTIC CORRESPONDENCE.

Reply to the *Dental Digest* . . . 279

NOTES AND COMMENTS . . . 282

CURRENT NEWS.

Important Notice . . . 285

United States Circuit Court, Southern District of New York . . . 286

Kentucky State Dental Association . . . 291

Illinois State Dental Society . . . 291

Lebanon Valley Dental Society . . . 292

Mississippi Valley Medical Association . . . 292

